# Effect of pupil dilation on biometric measurements and intraocular lens power calculations in schoolchildren

**DOI:** 10.1371/journal.pone.0203677

**Published:** 2018-09-13

**Authors:** Xiaogang Wang, Jing Dong, Maolong Tang, Xiaoliang Wang, Haitao Wang, Suhua Zhang

**Affiliations:** 1 Shanxi Eye Hospital, Shanxi, People’s Republic of China; 2 The First Hospital of Shanxi Medical University, Shanxi, People’s Republic of China; 3 Casey Eye Institute, Oregon Health and Science University, Portland, Oregon, United States of America; 4 School of Aeronautics and Astronautics Shanghai Jiao Tong University, Shanghai, P.R. China; Wenzhou Medical University Eye Hospital, CHINA

## Abstract

**Purpose:**

To investigate the effect of pupil dilation on ocular biometric parameters and intraocular lens (IOL) power calculation in schoolchildren using the Lenstar LS 900.

**Methods:**

One hundred forty eyes of 140 healthy schoolchildren were included in the analysis. Axial length (AL), central corneal thickness (CCT), aqueous depth (AD), anterior chamber depth (ACD), lens thickness (LT), flat keratometry (K), steep K, astigmatism, white-to-white (WTW), and iris/pupil barycenter distance were measured, before and after pupil dilation. Anterior segment length (ASL) was defined as the sum of ACD and LT, and lens position (LP) was defined as ACD plus half of the LT. The relative lens position (RLP) was defined as LP divided by AL. IOL power was calculated using the eight formulas (Hill-RBF, Barrett, Haigis, Hoffer Q, Holladay, Olsen, SRK II, and SRK/T) integrated in the Lenstar LS 900. Parameters before and after pupil dilation were compared.

**Results:**

AL, AD, ACD, LT, ASL, LP, RLP, flat K, iris barycenter distance, pupil barycenter distance, and PD differed significantly after pupil dilation (P < 0.001 in all cases), as compared to before dilation. The Olsen formula demonstrated significant differences in the magnitude of astigmatism (P = 0.010) and IOL power (P = 0.003) after pupil dilation. Using the different formulas, 23.6–40.7% of participants had IOL power changes of more than 0.50 diopters, while 0.7–1.4% had IOL changes of more than 1.0 diopter after pupil dilation.

**Conclusions:**

Dilated and undilated pupil size affected the Lenstar LS 900 measurement of some ocular biometric parameters, and pupil dilation led to IOL power changes exceeding 0.50 diopters with a high percentage (from 23.6% to 40.7%) in schoolchildren, which should be noticed in clinical practice.

## Introduction

Since its introduction in 1999 with the development of IOLMaster^™^, non-invasive optical biometry has increasingly been used by clinicians to determine ocular parameters and intraocular lens (IOL) power [1]. The Lenstar LS 900 (Haag-Streit Diagnostics, Köniz, Switzerland) became available in 2008. Unlike the IOLMaster, which uses partial coherence interferometry, the Lenstar LS 900 is based on optical low-coherence reflectometry. The device can measure axial length (AL), anterior chamber depth (ACD), and lens thickness (LT), among other parameters, in a single scan [2]. The latest Lenstar software (version 2.5.2) includes three new formulas (Hill-RBF, Barrett, and Olsen formulas), in addition to the five standard integrated formulas (Haigis, Hoffer Q, Holladay, SRK II, and SRK/T), providing cataract surgeons with more precise target refraction values.

One previous study has reported that pupil size may affect accommodation, convergence, and the accommodative convergence/accommodation (AC/A) ratio [3]. Moreover, several studies have investigated the influence of pupil dilation or cycloplegia on biometric parameters (AL, ACD, and keratometric values) and IOL power calculation (using the SRK/T, Holladay 1, Hoffer Q, Haigis formula) by the Lenstar LS 900 or IOLMaster [4–8].

Few studies have used optical biometry to compare pre- and post- pupil dilation biometric parameters, such as iris/pupil barycenter distance, central corneal thickness (CCT), and IOL power values determined by the Hill-RBF, Barrett, and Olsen formula, etc. Therefore, in the present study, we investigated the effect of pupil dilation on all biometric parameters. We also evaluated whether pupil dilation affected IOL power calculation based on the eight formulas integrated in the Lenstar LS 900.

## Methods

### Study population

This observational study was performed at the Shanxi Eye Hospital (Taiyuan, China) from October 2017 to December 2017 (S2 File). The research protocols were approved by the institutional review board of the Shanxi Eye Hospital and were carried out in accordance with the Declaration of Helsinki. The study was explained to the subjects’ legal guardians, who provided written informed consent.

One-hundred-and-forty eyes from 140 healthy schoolchildren (62 boys, 78 girls) were finally included in this study. The inclusion criteria were: best-corrected visual acuity (BCVA) better than 16/20, refractive error less than 4 spherical diopters (D), normal slit-lamp and fundoscopy examination results, intraocular pressure (IOP) less than 21 mmHg, and no history of ocular or systemic corticosteroid use. The exclusion criteria were detectable ocular diseases, previous ocular surgery, use of contact lenses, and use of medicated eye drops (steroid, IOP-lowering, or dry eye-related eye drops).

### Data acquisition

The biometry procedures using the Lenstar LS 900 (ver. 2.5.2, Haag-Streit AG, Koeniz, Switzerland) has been described previously [9]. Briefly, the subjects placed their chin on a chin rest, pressed their forehead against a forehead strap, and aligned the investigated eye to the visual axis by means of a central fixation target. During the examination, the participants were asked to fixate on the internal light of the device or the target, and the device was focused based on the image of the eye on the monitor. Before image capture, the participants were asked to blink to create an optically smooth tear film over the cornea. Measurements disrupted by blinking or unstable fixation were excluded from the final analyses. Three consecutive measurements per eye were automatically obtained before and after pupil dilation using tropicamide phenylephrine eye drops (Mydrin-P, Santen Oy., Tampere, Finland). In each subject, all pre- and post-dilation measurements were taken within 1 hour on the same day. According to the manufacturer’s calibration guidelines for measurements, the function check runs for the first time when the instrument is commissioned. Subsequent zero adjustment intervals (1 week) will be specified by the software. The same experienced examiner (X.G.W.) performed all the function check and examinations.

### Ocular biometric parameters

Biometric parameters may differ after pupil dilation, and these differences may influence IOL power calculation, especially when using 4^th^ generation or later formulas. Such formulas use more biometric parameters, including AL, ACD, LT, white-to-white (WTW), etc., to calculate IOL power.

**Fig 1:**
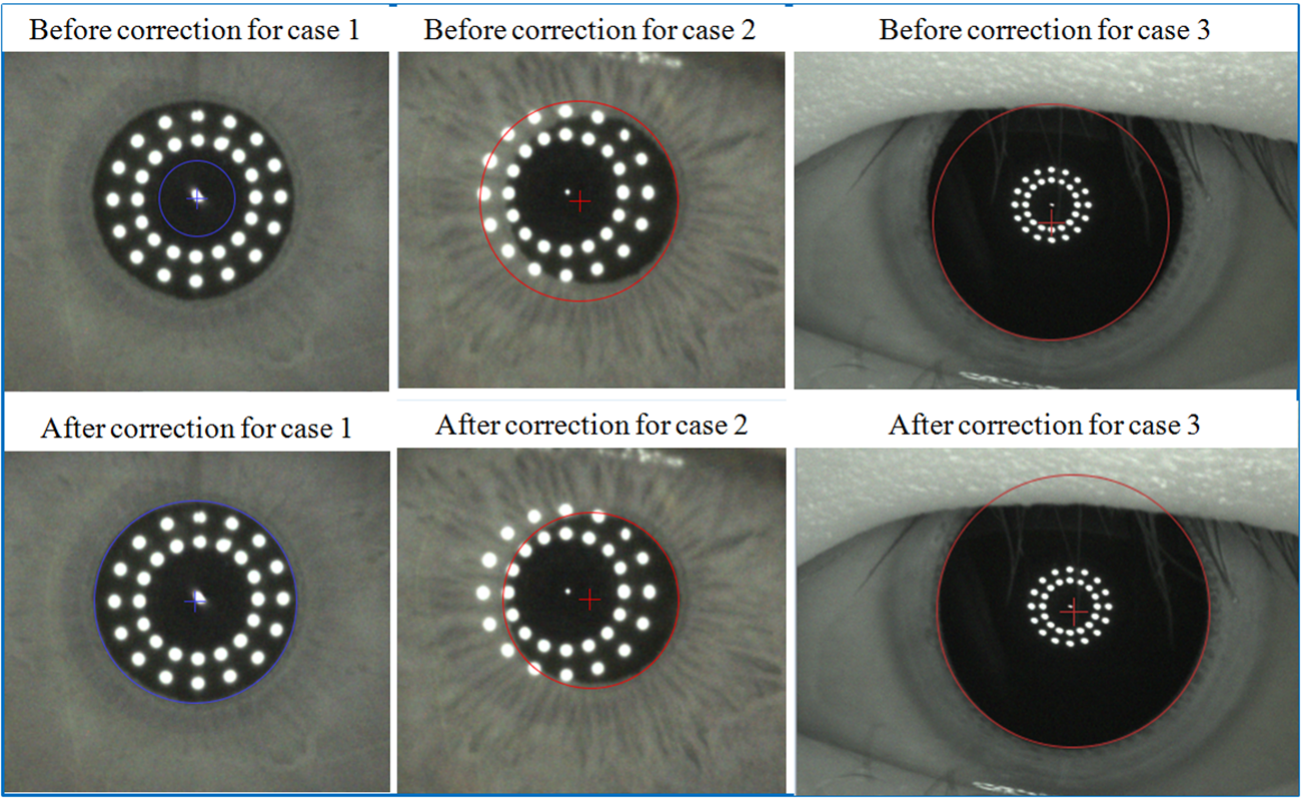
Three cases in which the pupil margins were inaccurately drawn despite valid measurements. In these three cases, the pupil margins were not appropriately defined for measuring pupil diameter (PD). In case 1, no PD or pupil barycenter data were obtained. After correction, the PD was 2.77 mm and the pupil barycentric coordinates were (-0.05, -0.02). In case 2, the default PD and pupil barycentric coordinates were 2.74 mm and (0.18, -0.12), respectively. After correction, the corresponding data were 2.39 mm and (0.32, -0.11). In case 3, the default PD and pupil barycentric coordinates were 7.79 mm and (-0.03, -0.59), respectively. After correction, the corresponding data were 8.54 mm and (0.09, -0.15).

The Lenstar LS 900 system automatically determined AL, CCT, aqueous depth (AD), ACD, LT, flat keratometry (K), steep K, astigmatism, pupil diameter (PD), WTW, the iris barycentric coordinates, and the pupil barycentric coordinates before and after pupil dilation. In the automatic measurements of PD and pupil barycentric coordinates, the pupil margins were redefined in the final data, as shown in Fig 1. There were no indications that any of these measurements were invalid. To calculate the iris and pupil barycentric coordinates, which are defined relative to the corneal apex, we measured the actual distance between the corneal apex and the iris center (iris barycenter distance [IBD]) or pupil center (pupil barycenter distance [PBD]). Anterior segment length (ASL) was defined as the sum of the ACD and LT, while the lens position (LP) was calculated as the sum of the ACD and half of the LT. The relative lens position (RLP) was defined as LP divided by AL [10,11].

### Intraocular lens power calculations

The internal software of the Lenstar LS 900 (version 4.2.1) was used to calculate IOL power. We set the target refraction to zero to observe whether pupil dilation affected IOL power calculation. To calculate the ideal IOL power, we chose the AR40e (AMO) as the target IOL model for each eye. All default formulas and their related parameters were used to calculate IOL power, as shown below. In the Hill-RBF formula, the A-constant was 118.7. In the Barrett and Holladay formulas, the lens factors were 1.73 and 1.63, respectively. In the Haigis formula, the A0, A1, and A2 values were -2.420, 0.157, and 0.288, respectively. In the Hoffer Q formula, the peripheral ACD was 5.41. In the Olsen formula, the ACD, N, LT, AR, PR, and C were 4.50, 1.47000, 0.90, 11.000, 16.000, and 0.37, respectively. In the SRK II and SRK/T formulas, the A-constants were 118.80 and 118.70, respectively.

### Statistical analyses

Statistical analyses were performed using commercial software (SPSS ver. 13.0; SPSS, Inc., Chicago, IL, USA). To compare the biometric parameters and IOL power before and after pupil dilation, paired two-tailed t-tests were performed. For all tests, the significance level was set at 5%.

## Results

One-hundred-forty-four healthy Chinese schoolchildren (144 eyes) were enrolled in this study (S1 File). Four eyes were excluded due to frequent blinking during the examination. Ultimately, 140 subjects (140 eyes) were included in the analysis: 62 boys (44%, 62 eyes) and 78 girls (56%, 78 eyes). Their mean age was 10 ± 2 years (range: 7–18 years).

Table 1 summarizes the mean ocular biometric parameters and IOL power values. Compared to pre-dilation, the AL, CCT, AD, ACD, ASL, LP, magnitude of astigmatism, iris distance, pupil distance, and PD were around 0.01 ± 0.02 mm, 14±6 μm, 0.06 ± 0.07 mm, 0.07 ± 0.07 mm, 0.02 ± 0.03 mm, 0.05 ± 0.04 mm, 0.06 ± 0.26 D, 0.10 ± 0.27 mm, 0.08 ± 0.18 mm, 4.18 ± 1.03 mm higher after pupil dilation, respectively (all P < 0.05). Conversely, the LT, RLP, flat K of post-dilation were 0.05 ± 0.08 mm, 0.002 ± 0.001, 0.04 ± 0.12 D lower than that of pre-dilation, respectively (all P < 0.05). The Olsen formula also revealed significantly higher differences in IOL power (P = 0.003) after pupil dilation. However, the other seven formulas demonstrated no statistical difference in IOL power after pupil dilation (all P > 0.05).

**Table 1:** Summary of the mean ocular biometric measurements and IOL power calculations before and after pupil dilation.

	Before pupil dilation (n = 140)	After pupil dilation (n = 140)	Mean Difference	P-value*
AL (mm)	24.07 ± 1.54	24.09 ± 1.54	-0.01 ± 0.02	**< 0.001**
CCT (μm)	545 ± 31	559 ± 31	-14 ± 6	**< 0.001**
AD (mm)	3.17 ± 0.26	3.22 ± 0.23	-0.06 ± 0.07	**< 0.001**
ACD (mm)	3.71 ± 0.26	3.78 ± 0.24	-0.07 ± 0.07	**< 0.001**
LT (mm)	3.38 ± 0.19	3.34 ± 0.15	0.05 ± 0.08	**< 0.001**
ASL (mm)	7.10 ± 0.27	7.12 ± 0.27	-0.02 ± 0.03	**< 0.001**
LP (mm)	5.40 ± 0.25	5.45 ± 0.24	-0.05 ± 0.04	**< 0.001**
RLP	0.225 ± 0.013	0.227 ± 0.014	0.002± 0.001	**< 0.001**
K flat (D)	42.89 ± 1.40	42.85 ± 1.40	0.04 ± 0.12	**< 0.001**
K steep (D)	44.16 ± 1.63	44.18 ± 1.61	-0.02 ± 0.24	0.404
Magnitude of astigmatism (D)	1.27 ± 0.71	1.32 ± 0.71	-0.06 ± 0.26	**0.010**
Axis of astigmatism (degree)	88 ± 17	88 ± 16	-0.2 ± 14	0.865
WTW (mm)	11.98 ± 0.44	11.98 ± 0.45	0 ± 0.20	0.902
IBD (mm)	0.38 ± 0.21	0.48 ± 0.27	-0.10 ± 0.27	**< 0.001**
PD (mm)	3.84 ± 0.79	8.02 ± 0.77	-4.18 ± 1.03	**< 0.001**
PBD (mm)	0.19 ± 0.12	0.27 ± 0.15	-0.08 ± 0.18	**< 0.001**
**IOL power**				
Hill-RBF (D)	19.8 ± 4.7	19.8 ± 4.6	0.01 ± 0.28	0.548
Hill-RBF SE (D)	-0.16 ± 0.09	-0.16 ± 0.09	0 ± 0.13	0.655
Barrett (D)	19.4 ± 4.6	19.3 ± 4.6	0.02 ± 0.28	0.448
Barrett SE (D)	-0.01 ± 0.10	-0.01 ± 0.11	0 ± 0.14	0.716
Haigis (D)	19.6 ± 4.6	19.6 ± 4.5	0 ± 0.33	0.899
Haigis SE (D)	± 0.10	0 ± 0.11	0 ± 0.16	0.752
HofferQ (D)	19.3 ± 4.9	19.3 ± 4.8	0.03 ± 0.32	0.243
HofferQ SE (D)	-0.01 ± 0.10	-0.01 ± 0.09	0 ± 0.13	0.875
Holladay (D)	19.3 ± 4.7	19.3 ± 4.7	0.01 ± 0.28	0.764
Holladay SE (D)	0.01 ± 0.10	-0.01 ± 0.09	0.01 ± 0.13	0.203
Olsen (D)	19.2 ± 4.7	19.3 ± 4.7	-0.08 ± 0.31	**0.003**
Olsen SE (D)	0.01 ± 0.10	0 ± 0.11	0.01 ± 0.14	0.326
SRK II (D)	19.4 ± 4.0	19.4 ± 4.0	0.03 ± 0.24	0.118
SRK II SE (D)	0 ± 0.12	0 ± 0.13	-0.01 ± 0.16	0.513
SRK/T (D)	19.3 ± 4.5	19.2 ± 4.5	0.01 ± 0.25	0.495
SRK/T SE (D)	0 ± 0.10	0 ± 0.10	0.01 ± 0.12	0.309

Note: AD = aqueous depth; ACD = anterior chamber depth; AL = axial length; ASL = anterior segment length; CCT = central corneal thickness; D = diopter; IOL = intraocular lens; IBD = iris barycenter distance; K = keratometry; LP = lens position; LT = lens thickness; PBD = pupil barycenter distance; PD = pupil diameter; RLP = relative lens position; SE = spherical equivalent; WTW = white-to-white distance

*Paired-sample t-test

**Fig 2:**
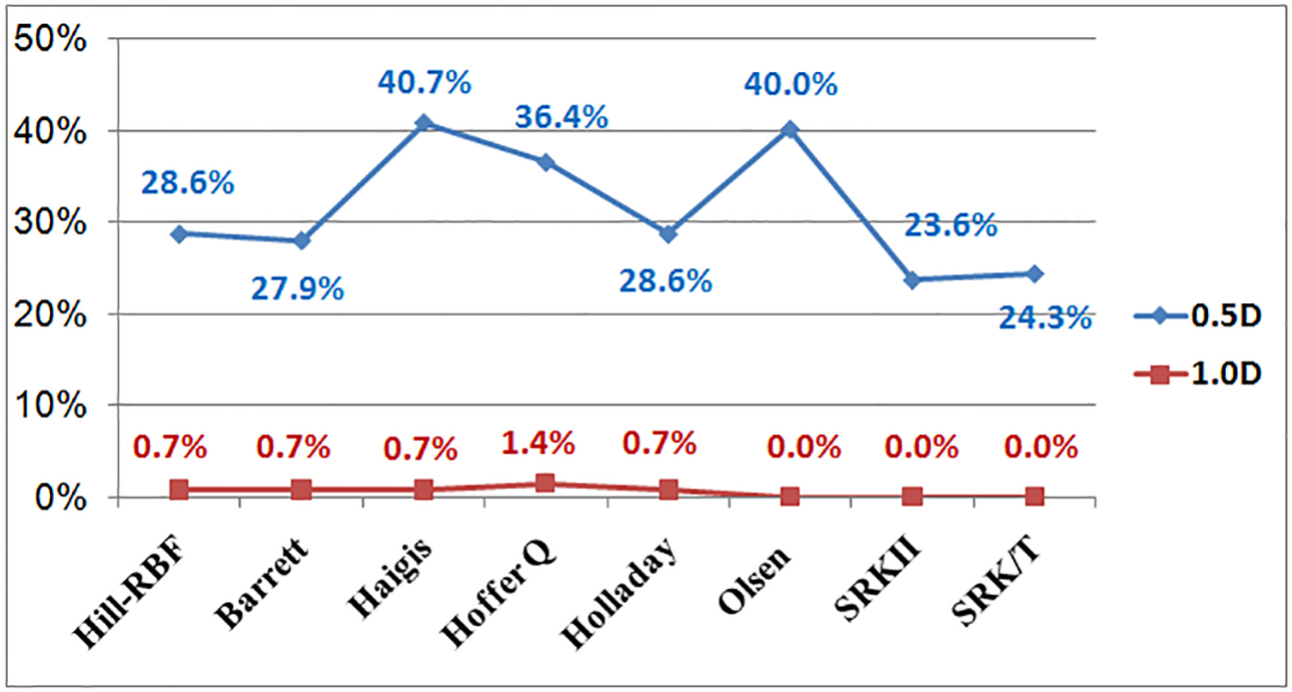
Percentage of participants whose intraocular lens power changed by more than 0.50 diopters and by more than 1.0 diopter after pupil dilation, as calculated using various formulas.

For IOL power fluctuation before and after pupil dilation, the Table 2 and Fig 2 demonstrate that SRKII formula has 33 eyes (the lowest percentage, 23.6%) and Haigis formula has 57 eyes (the highest percentage, 40.7%) whose IOL power changed by more than 0.50 D. Moreover, Olsen, SRKII, SRK/T formulas show no eye (the lowest percentage, 0%) and Hoffer Q formula shows 2 eyes (the highest percentage, 1.4%) whose IOL power changed by more than 1.0 D.

**Table 2:** Number of participants whose intraocular lens power changed by more than 0.50 diopters and by more than 1.0 diopter after pupil dilation, as calculated using different formulas.

	Hill-RBF	Barrett	Haigis	Hoffer Q	Holladay	Olsen	SRK II	SRK/T
≥ 0.5 D	40	39	57	51	40	56	33	34
≥ 1.0 D	1	1	1	2	1	0	0	0

D = diopter

## Discussion

Pupil dilation is essential for detailed clinical examination and is sometimes performed before biometry. Moreover, dilating the pupil may aid AL measurement in eyes with dense nuclear and posterior subcapsular cataracts, which complicate biometric measurement with the Lenstar or IOLMaster [12]. Previous research has demonstrated that AL measurement using the Lenstar has good reliability, and that pupil dilation does not influence AL measurement or IOL power calculation when using the SRK/T formula [4]. However, it is unclear whether pupil dilation influences other biometric parameters or IOL power determined using other formulas.

The present study indicated that pupil dilation influences all ocular biometric measurements other than steep K, astigmatism axis, and WTW; AL, CCT, AD, ACD, LT, ASL, LP, RLP, flat K, astigmatism magnitude, IBD, and PBD were all significantly affected by pupil dilation. Moreover, the pre-dilation IOL power was -0.08 ± 0.31 D lower than the post-dilation values determined with the Olsen formula (P = 0.003) but not the other seven formulas integrated into the Lenstar LS 900 (P > 0.05). However, based on the different IOL power formulas, 23.6–40.7% of participants had IOL power changes of more than 0.50 diopters, and 0.7–1.4% had IOL changes of more than 1.0 diopter.

Inconsistent with previous studies using IOLMaster or Lenstar, which found that cycloplegia had no significant effect on AL measurements [4–8]. In the current study, the pre-dilation AL measurements were -0.01 ± 0.02 mm lower than the post-dilation values (P < 0.001). A similar but not significant tendency had been found in a previous study involving the Lenstar LS 900 [4]. A 0.01-mm difference in AL results in an IOL power difference of around ± 0.028 diopter, which is not clinically significant [13]. In the present study, the AL—as measured from the anterior cornea to the retinal pigment epithelium—varied, probably because the CCT increased by about 14 μm as a result of temporary corneal swelling one hour after pupil dilation, which has been demonstrated in previous studies [14,15]. Moreover, the potential instrument bias, which may influence the accuracy of measurement, should also be considered when interpreting the current result.

Using the Pentacam^™^, Atalay et al. found that post-dilation CCT measurements were significantly lower than pre-dilation values in normal eyes and eyes with pseudoexfoliation syndrome [16]. In contrast, our study showed that the post-dilation CCT was 14 μm thicker than the pre-dilation measurement. This may be due to the different imaging technology used—the Lenstar uses low-coherence reflectometry, while the Pentacam employs Scheimpflug imaging.

We found that the AD and ACD values obtained with the Lenstar after pupil dilation were statistically significantly thicker than those before dilation, corroborating previous studies [4,8,17,18]. Moreover, the post-dilation LT was about 0.05 ± 0.08 mm thinner than the pre-dilation value. It is likely that these changes occur because pupil dilation affects accommodation, and the consequent posterior movement and flattening of the crystalline lens increase the ACD [17]. The ASL, LP, and RLP were interdependent and were used to study the effect of the lens on the anterior chamber. In addition, these three factors were dependent on LT, ACD, and lens position, which is maintained by the zonulae and ciliary muscle [19]. Therefore, post-dilation differences in LT and ACD may contribute to differences in ASL, LP, and RLP.

Our results are inconsistent with those of previous studies, which found a statistically significant change in flat-K readings after pupil dilation [4,8]. We speculate that mydriatics cause corneal epithelial metabolism changes, which may, in turn, affect the reproducibility of K readings. Changes in accommodation status may also lead to differences in flat K readings [20]. Importantly, astigmatism was calculated using the steep K and flat K values in the present study. Thus, the above-mentioned reasons may also explain the magnitude of the difference in astigmatism found in the present study. Specifically, the pre-dilation magnitude of astigmatism was about -0.06 ± 0.26 diopter lower than the post-dilation magnitude. This finding is important to consider when using toric IOL implantations to manage corneal astigmatism.

No significant difference in WTW was found after pupil dilation in the present study. This finding differed from that of Huang et al., who found a 0.1-mm difference [8]. Both studies used the same biometry system, and the discrepancy may therefore be due to examiner manipulation and imaging artifacts.

The present study found differences in PBD and IBD after pupil dilation, confirming that the pupil center and iris center shift as the pupil dilates, as reported in a previous study [21]. Participants with relatively large pupil center and iris center shifts, which are associated with IOL decentration, may look through the diffractive rings rather than through the central optical zone. This can cause uncomfortable photic phenomena (glare, coma, haloing) and may ultimately decrease postoperative visual function [22]. Therefore, our results provide a basis for more detailed and accurate counseling and planning, allowing the optimal IOL selection.

Four of the IOL formulas—SRK II, SRK/T, Holladay, and Hoffer Q—are based on only the AL and keratometry reading. The Haigis formula requires three parameters: AL, ACD, and keratometry reading. The Barrett formula uses five parameters to calculate IOL power: AL, keratometry reading, ACD, LT, and WTW. In the Olsen formula, there are six input parameters: AL, ACD, keratometry reading, LT, patient age, and CCT [23]. Unlike these static theoretical formulas, the Hill-RBF formula, which employs pattern recognition and multi-dimensional data interpolation, is a self-validating method that uses the radial basis function to calculate IOL power [24]. In the present study, the mean IOL power difference after pupil dilation, as calculated by the Lenstar, varied between -0.08 and 0.02 diopter, depending on which of the eight formulas was used. This finding is consistent with that of a previous investigation that used the SRK/T formula in the same biometry system [4]. Only the Olsen formula showed about -0.08 ± 0.31 diopter significant difference in pre- vs post-dilation. This may attribute to more input parameters, which were all affected by pupil dilation except patient age in this study, used for Olsen formula, as compared to the other seven formulas.

Using the different IOL power formulas, 23.6–40.7% of participants had IOL power changes of more than 0.50 diopters, and 0.7–1.4% had IOL changes of more than 1.0 diopter, after pupil dilation. This indicates that some formulas are more sensitive to biometric changes that follow pupil dilation. Furthermore, this difference may in turn be caused by significant differences in AL, ACD, flat K, LT, and CCT, which are all used in the IOL power calculation formulas.

The current study had several limitations. Firstly, previous studies have shown that subjects with different ethnic backgrounds have different ocular biometry [25,26]. This cross-sectional study involved only Chinese subjects and therefore cannot be generalized to individuals of other ethnicities. Secondly, we did not divide the subjects into AL subgroups in the present study, which may have affected the results. Thus, clinicians must interpret these results with caution, even when using the same biometry system. Thirdly, instrument bias originating from temperature/humidity variation in the examination room, power supply voltage drift may affect the measurement accuracy. Therefore, we put the device in an appropriative room and do the function check each week to lower the potential influence of the above-mentioned factors. As a final limitation, the present study only enrolled schoolchildren, and thus may not be applicable to individuals of other ages. Despite these limitations, this prospective study provides information that is useful for clinical practice.

In conclusion, pupil dilation affected some ocular biometric parameters, as measured using the Lenstar LS 900. Notably, a high percentage of participants showed IOL power changes of more than 0.50 diopters after pupil dilation.

## Supporting information

S1 File Data set. (XLSX)

S2 File STROBE research checklist. (DOC)
